# Cancer survival prediction based on soft-label guided contrastive learning and global feature fusion

**DOI:** 10.1093/bioinformatics/btaf552

**Published:** 2025-10-01

**Authors:** Huiying Jiang, Wenlan Chen, Fei Guo, Cheng Liang

**Affiliations:** School of Information Science and Engineering, Shandong Normal University, Jinan 250358, China; School of Computer Science and Engineering, Central South University, Changsha 410083, China; School of Computer Science and Engineering, Central South University, Changsha 410083, China; School of Information Science and Engineering, Shandong Normal University, Jinan 250358, China

## Abstract

**Motivation:**

The high complexity and heterogeneity of cancer pose significant challenges to personalized treatment, making the improvement of cancer survival prediction accuracy crucial for clinical decision-making. The integration of multi-omics data enables a more comprehensive capture of multi-layered information in complex biological processes. However, existing survival analysis models still face limitations in accurately extracting and effectively integrating the unique and shared information from multi-omics data.

**Results:**

In this article, we propose a novel prediction model for cancer survival based on soft-label guided contrastive learning and global feature fusion, namely SLCGF. Our model first extracts paired feature representations for each omics using Siamese encoders. We then perform intra-view and inter-view contrastive learning simultaneously, employing a neighborhood-based paradigm to enhance feature discrimination and alignment across omics. To ensure reliable neighbor retention and improve model robustness, we treat the affinities between samples and their high-order neighbors as soft labels to guide the contrastive learning process at both levels. In addition, we adopt a global self-attention mechanism to obtain the unified representation for cancer survival prediction, where the cross-omics connections are fully exploited and complementary information is adaptively integrated. We comprehensively evaluate the performance of our model on 13 cancer multi-omics datasets, and the experimental results demonstrate its superiority over existing approaches.

**Availability and implementation:**

Source code is available at https://github.com/LiangSDNULab/SLCGF.

## 1 Introduction

Cancer, due to its high complexity and significant heterogeneity, exhibits diverse molecular mechanisms and clinical characteristics across patients (Dagogo-Jack and Shaw 2018, Pan *et al.* 2024). This variability poses a substantial challenge in providing precise treatment options tailored to each individual. Recently, survival analysis has been widely applied in the biomedical field (Cui *et al.* 2024). It aims to predict the time elapsed from a patient’s diagnosis or treatment condition to events such as death, recurrence, or disease progression. Accurate survival prediction for cancer patients can assist clinicians in making more informed decisions and further facilitating the provision of appropriate and effective treatment guidance (Huang *et al.* 2020).

With the rapid advancement of sequencing technologies, the quantity and variety of omics data have significantly expanded, enabling the acquisition of more comprehensive biological information (Chai *et al.* 2021). The availability of omics data, such as gene expression and microRNA expression, has made it possible to apply computational models to survival analysis (Albaradei *et al.* 2021). Statistical models, as an important tool in survival analysis, can be broadly classified into non-parametric (Zhang 2016), parametric (Cox *et al.* 2007), and semi-parametric (Müller and Quintana 2004) methods, based on their assumptions about the distribution of survival time and the way they model covariates. Non-parametric methods analyze survival data without assuming a specific distribution, with the Kaplan–Meier method (Kaplan and Meier 1958) being a common example. Parametric methods assume that the survival time of cancer patients follows a specific probability distribution, and parameters are estimated based on this assumption (Nardi and Schemper 2003). Semi-parametric methods do not make assumptions about the underlying distribution of survival time, but instead analyze survival data by modeling the impact of covariates. These method typically combine the advantages of both parametric and non-parametric approaches, and are particularly effective when handling the Cox proportional hazards model (Cox 1972) and its extensions (Wiegrebe *et al.* 2024). For instance, CoxBoost (Binder *et al.* 2009) is an enhanced method based on the Cox model, which iteratively optimizes the partial log-likelihood function and incorporates regularization techniques to reduce overfitting. Although traditional statistical methods have driven the development of survival analysis, their reliance on linear assumptions and manual feature engineering often limits their ability to capture complex nonlinear interactions in high-dimensional omics data. To overcome this challenge, deep learning methods (Vale-Silva and Rohr 2021, Hou *et al.* 2023) have emerged. Initially, deep learning methods are widely applied to single-omics data analysis, with early research primarily focusing on feature-based approaches that extract important features from a single type of omics data and use deep learning models for survival prediction. For example, DeepSurv (Katzman *et al.* 2018) integrates deep neural networks with the classical Cox proportional hazards model, leveraging deep learning to automatically learn nonlinear features from the input omics data and using them for survival prediction. In Cox-nnet (Ching *et al.* 2018), the hidden nodes of the neural network are used for downscaling high-dimensional data. With neural networks, complex high-dimensional input data are compressed into a low-dimensional space of potential features, and these downscaled features can capture key information in the data. VAECox (Kim *et al.* 2020) addresses the issue of data scarcity by leveraging transfer learning and fine-tuning, thereby preventing overfitting in deep learning models. SurvNet (Wang *et al.* 2021), by introducing the input reconstruction module and context gating mechanism, addresses the issue of missing values and reduces the gap between survival classification and Cox regression in prognosis prediction.

However, as the limitations of single-omics data became apparent, attention shifted toward multi-omics approaches, which integrate multiple types of omics data to capture a more comprehensive view of biological processes and improve the accuracy of prognosis prediction (Jiang *et al.* 2024). For instance, MDNNMD (Sun *et al.* 2018) leverages three differently weighted deep neural networks to integrate information from gene expression profiles, CNA profiles, and clinical data, enabling survival prediction for breast cancer. OmicsGAN (Ahmed *et al.* 2022) introduces a generative adversarial network approach that integrates information from diverse omics datasets and interaction networks, significantly enhancing survival prediction performance. OmicsNMF (Ansari *et al.* 2024) integrates Generative Adversarial Networks and Non-Negative Matrix Factorization to address the issue of missing samples, thus improving data integration and predictive accuracy. CAGCL (Palmal *et al.* 2024) enhances feature embedding through graph contrastive learning, enabling the identification of subtle differences and similarities between samples. Additionally, a cross-attention framework is proposed to fuze features from different omics for breast cancer survival prediction. FGCNSurv (Wen and Li 2023) utilizes a GCN to fuze both single-omics and cross-omics features, combining multiple omics maps into a dual fusion map to capture advanced features for survival prediction. MMOSurv (Wen and Li 2025) proposes a meta-learning framework for few-shot survival analysis. It learns survival model parameters from related cancer multi-omics data and adjusts them with a small number of samples to adapt to the target cancer task, improving survival prediction with minimal data.

Despite the strong potential of multi-omics approaches in cancer survival prediction, existing models still suffer from the following limitations. Existing methods for omics data may fail to fully explore the underlying relationships between samples, resulting in the inability to extract sufficiently discriminative feature representations. Furthermore, in the multi-omics feature fusion phase, many methods directly concatenate latent multi-omics features to form fused representations, thereby overlooking the complex relationships across omics types. To address these limitations, we propose a novel cancer survival prediction model called SLCGF based on soft-label guided contrastive learning and global feature fusion. In our model, paired feature representations for each omics are first extracted using Siamese encoders. Next, we perform both intra-view and inter-view contrastive learning simultaneously, employing a neighborhood-based paradigm to enhance feature discrimination and alignment across omics. Specifically, to ensure the stability of the neighborhood structure, we treat the similarity between samples and their higher-order neighbors as soft labels to guide the contrastive learning process. Additionally, we incorporate a global self-attention module that effectively captures global information, enabling adaptive generation of a unified feature representation for cancer survival prediction. We conducted extensive experiments on 13 cancer datasets to validate the effectiveness of our model.

## 2 Materials and methods

**Figure 1.**
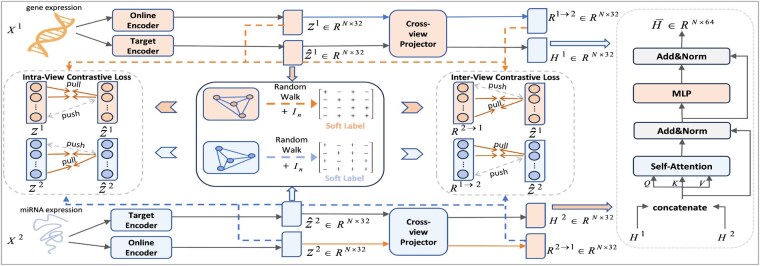
The overall workflow of SLCGF. The Siamese encoder and cross-view projector extract view-specific and consistency information. Soft-label guided contrastive learning makes similar samples closer and dissimilar samples farther apart based on the soft labels generated by random walks. The global feature fusion module captures global relationships to form a fused representation.

We propose a method based on soft-label guided contrastive learning and global feature fusion for cancer survival prediction. Our model effectively captures the specific information as well as shared representations from different omics and performs efficient global feature fusion. The framework of SLCGF is shown in Fig. 1 and Table 1 summarizes key notations used in the article. In the following, we will provide a detailed explanation of each component in our model.

**Table 1.** Notations used throughout this study.

Notations	Definition
$\left\{X^{1},X^{2},\ldots ,X^{V}\right\}$	Multi-omics dataset with *V* omics types
$X^{v}\in R^{N\times D_{v}}$	Data matrix of the *v*th omics for *N* patients
$x_{i}^{v}\in R^{D_{v}}$	The *i*th sample of the *v*th omics
$z_{i}^{v}$, ${\hat{z}}_{i}^{v}\in R^{d_{r}}$	Output features of Siamese encoder
$R_{i}^{v\to m}\in R^{d_{m}}$	Output features of cross-view projector
$G^{v}\in R^{N\times N}$	Affinity matrix
$I_{n}\in R^{N\times N}$	Identity matrix
$M^{v^{\left(s\right)}}\in R^{N\times N}$	Multi-step transition matrix
$P^{v}\in R^{N\times N}$	Soft label matrix
$\bar{H}\in R^{N\times D}$	Final fused features

### 2.1 Datasets

Thirteen cancer multi-omics datasets are selected to evaluate the performance of the method. Among them, 10 cancer datasets are downloaded from The Cancer Genome Atlas (TCGA) (Zhu *et al.* 2014), including AML, Breast, Colon, GBM, Kidney, Liver, Lung, Melanoma, Ovarian, Sarcoma. An integrated dataset combining eight of the 10 aforementioned cancer datasets generated from the same sequencing platform is then constructed to evaluate the model performance on large-scale data. Moreover, a more challenging dataset LUAD comprising four omics types, i.e. gene expression, miRNA expression, copy number variation (CNV), and DNA methylation provided by MLOmics (Yang *et al.* 2025) is used to validate the extendability of each model. Lastly, we also adopt a Kidney Renal Papillary Cell Carcinoma (KIRP) dataset provided by MLOmics (Yang *et al.* 2025) as an independent test set to assess the generalization ability of all models, where this dataset is excluded from all training procedures and solely used for external validation. For each dataset except LUAD, we use the mRNA expression and miRNA expression data as model input. Specifically, for gene expression data, we select the top 2000 genes with the highest variance; for miRNA expression data, we remove miRNA features with zero variance. As for the LUAD dataset, we similarly select the top 2000 features with the highest variance for the CNV and DNA methylation data. All omics data are normalized to have zero mean and unit variance before being input into the model. Detailed information about all cancer datasets is provided in Table 1, available as supplementary data at *Bioinformatics* online.

### 2.2 View-specific encoder and cross-view projector module

Considering the characteristics and information heterogeneity of different omics, we design view-specific encoder and a cross-view projector to enhance consistency learning between views while preserving the unique information of each view.

#### 2.2.1 View-specific Siamese encoders

We first generate low-dimensional feature representations for each omics using the Siamese encoder, which is designed to learn the shared underlying structure between different omics by comparing their pairwise similarities. The Siamese network consists of two identical network structures: an online encoder and a target encoder. The online encoder directly participates in the training process, and its parameters $\theta_{z}^{v}$ are updated through backpropagation according to the loss function. In contrast, the parameters $\theta_{t}^{v}$ of the target encoder are updated using the Exponential Moving Average (EMA) of the online encoder. The use of EMA helps stabilize the model by smoothing the updates, which leads to more consistent and reliable parameter updates (Morales-Brotons *et al.* 2024). The parameters of the target encoder are updated as follows:


(1) $$\theta_{t}^{v}\leftarrow \alpha \theta_{t}^{v}+(1-\alpha )\theta_{z}^{v}\;$$

Here, α is a smoothing coefficient to ensure that the parameter changes in the target encoder are slow and smooth.

We feed each omics dataset separately into the online encoder network $f_{z}^{v}(\cdot )$ and the target encoder $f_{t}^{v}(\cdot )$ to learn low-dimensional features, i.e. respectively:


(2) $$\;z_{i}^{v}=f_{z}^{v}(x_{i}^{v};\theta_{z}^{v})$$

(3) $$\;{\hat{z}}_{i}^{v}=f_{t}^{v}(x_{i}^{v};\theta_{t}^{v})$$

#### 2.2.2 Cross-view projectors

During cross-view consistency learning, focusing solely on maximizing the consistency between the embedded features extracted by different view encoders may overlook the unique information contained within each view, leading to the loss of such information. Therefore, we propose a cross-view projector that avoids the forced alignment of features from all views into a shared common space, effectively preserving the unique information of each view. Specifically, we input the low-dimensional embedding $z_{i}^{v}$ obtained from the *v*th online encoder into the cross-view projector $R^{v\to m}(\cdot )$, a two-layer MLP network. The projector then projects $z_{i}^{v}$ into the embedding space of another view *m* (m≠v), producing the mapped embedding $R_{i}^{v\to m}$:


(4) $$\;R_{i}^{v\to m}=R^{v\to m}(z_{i}^{v};\phi )$$

where ϕ represents the parameters of cross-view projector.

### 2.3 Soft-label guided contrastive learning

To enhance the discriminative power of feature representations and the learning of correlations between different omics data, we introduce a soft-label guided contrastive learning approach. Specifically, we divide the contrastive loss function into two parts: intra-view contrastive loss and inter-view contrastive loss.

#### 2.3.1 Intra-view contrastive loss

Although the Siamese encoder can extract paired low-dimensional feature representations for each omics view, it may not fully capture the finer-grained and high-level semantic information within each view. To address this issue, we introduce intra-view contrastive learning to enhance the discriminative power of the feature representations. The goal of intra-view contrastive learning is to bring similar samples closer together in the embedding space within the same view, while pushing dissimilar samples further apart. To enhance the reliability of intra-view contrastive learning, we utilize high-order random walks (Lovász 1993) to capture the higher-order neighborhood relationships between samples. This allows us to incorporate the affinity between a sample and its higher-order neighbors as soft labels, guiding the contrastive learning process more effectively. To fully account for the unique characteristics of each view, we construct independent fully connected affinity graph for each view. Specifically, for the embedding representations ${\hat{z}}_{i}^{v}$ of each view, we use the heat kernel similarity (Wen 2020) to measure the similarity between two embeddings as follows:


(5) $$G_{i,j}^{v}=\text{exp}(\frac{-∥{\hat{z}}_{i}^{v}-{\hat{z}}_{j}^{v}{∥}^{2}}{\sigma })$$

where σ is a hyperparameter controlling the band width. The heat kernel similarity could effectively preserve local neighborhood structures through a smooth exponential decay mechanism, and thus provides a more accurate characterization of the semantic relationships between samples. More analysis regarding the effects of other similarity measures such as Euclidean distance and cosine similarity on the model performance can be found in Section 10, available as supplementary data at *Bioinformatics* online. We then normalize the rows of the affinity matrix $G^{v}$ to obtain the random walk transition matrix $M^{v}$ for each view. Then, after *s* steps of random walks, we obtain the multi-step transition matrix $M^{v^{\left(s\right)}}$, which reflects the high-order neighborhood relationships between nodes. We also integrate the identity matrix $I_{n}$ into the multi-step transition matrix $M^{v^{\left(s\right)}}$ to preserve the self-connections and the final soft label matrix $P^{v}$ can be expressed as:


(6) $$P^{v}=\gamma I_{n}+(1-\gamma )M^{v^{\left(s\right)}}$$

where γ is a hyperparameter that controls the balance between the identity matrix and the multi-step transition matrix. This soft label matrix assigns different weights to positive and negative samples in the contrastive learning process, allowing for a more fine-grained adjustment of the relationships between samples. Based on this, we next introduce the loss function for intra-view contrastive learning. Specifically, the contrastive learning loss function is guided by the soft label matrix $P^{v}$ and is defined as:


(7) $$L_{\mathrm{intra}}=-\underset{v=1}{\sum^{V}}\underset{i,j}{\sum^{N}}P_{i,j}^{v}\;\text{log}\;\frac{\;\text{exp}(\mathrm{sim}(z_{i}^{v},{\hat{z}}_{j}^{v})/\tau )}{\sum_{k=1}^{N}\;\text{exp}(\mathrm{sim}(z_{i}^{v},{\hat{z}}_{k}^{v})/\tau )}$$

where


(8) $$\mathrm{sim}(z_{i}^{v},{\hat{z}}_{j}^{v})=\frac{(z_{i}^{v}){\left({\hat{z}}_{j}^{v}\right)}^{T}}{\left||z_{i}^{v}||||{\hat{z}}_{j}^{v}|\right|}$$

here τ is the temperature coefficient, and $\mathrm{sim}(z_{i}^{v},{\hat{z}}_{j}^{v})$ represents the similarity between sample pairs, which is measured using cosine similarity. By minimizing this loss function, the model can effectively learn the sample features within each view, optimizing the embedding space.

#### 2.3.2 Inter-view contrastive loss

The cross-view projector decouples the feature learning between views from a shared space into view-specific spaces, thereby effectively preserving the unique information of each view. However, such decoupling alone may not be sufficient to fully explore the intrinsic associations between views. Therefore, to further promote consistency learning between views while preserving view-specific information, we introduce an inter-view contrastive loss. Specifically, we take the embedding $R^{v\to m}$ obtained from the cross-view projector of the *v*th view and the representation ${\hat{z}}^{m}$ extracted by the target encoder of the *m*th view as the input for inter-view contrastive learning. Moreover, we employ the same soft label matrix $p^{m}$ from the intra-view contrastive learning to guide the inter-view training. This strategy enhances the consistency of cross-view representation alignment and meanwhile mitigates the structural instability and information redundancy that may arise from reconstructing independent soft labels, as evidenced by the results in Section 11, available as supplementary data at *Bioinformatics* online. Based on this, the inter-view contrastive loss is defined as follows:


(9) $$L_{\mathrm{inter}}=-\underset{m=1}{\sum^{V}}\underset{v\neq m}{\sum^{V}}\underset{i,j}{\sum^{N}}P_{i,j}^{m}\;\text{log}\;\frac{\;\text{exp}(\mathrm{sim}(R_{i}^{v\to m},{\hat{z}}_{j}^{m})/\tau )}{\sum_{k=1}^{N}\;\text{exp}(\mathrm{sim}(R_{i}^{v\to m},{\hat{z}}_{k}^{m})/\tau )}$$

### 2.4 Global feature fusion module

To better integrate multi-omics information, we propose a global feature fusion approach in this section. We input the stable embedding ${\left\{{\hat{z}}^{v}\right\}}_{v=1}^{V}$ obtained from the target encoder into the cross-view projector to obtain the feature representation ${\left\{H^{v}\right\}}_{v=1}^{V}$. Then, we concatenate all the obtained feature representations to finally obtain $H\in R^{N\times d}$, as expressed in the following formula:


(10) $$H=[H^{1},H^{2},\ldots ,H^{V}]\;$$

However, the simple concatenation operation illustrated in the above equation struggles to capture the complex interactions among multiple omics. Inspired by the transformer architecture (Yan *et al.* 2023), we utilize a global feature fusion module to more effectively extract common semantics from multiple omics. Initially, we apply distinct linear mappings to *H* to obtain the corresponding query, key, and value, represented as $Q=HW_{Q}$, $K=HW_{K}$, and $V=HW_{V}$, where $W_{Q}\in R^{d\times d_{k}}$, $W_{K}\in R^{d\times d_{k}}$, $W_{V}\in R^{d\times d_{k}}$ are the weights of the corresponding mapping layers, respectively. The attention score matrix is calculated as $S=\mathrm{softmax}(\frac{QK^{T}}{\sqrt{d_{k}}})$, where $1/\sqrt{d_{k}}\;$ serves as a scaling factor. The final output of the scaled dot-product attention layer is:


(11) T=SV

Next, to stabilize training and prevent gradient vanishing, we apply residual connections and layer normalization to the output of the attention layer, resulting in the intermediate output $\tilde{H}=\mathrm{LayerNorm}(H+T)$. Then, we further capture global features through the MLP layer. Finally, we obtain the fusion representation through residual connection and layer normalization once again:


(12) $$\bar{H}=\mathrm{LayerNorm}(\tilde{H}+\mathrm{MLP}(\tilde{H}))$$

where LayerNorm(·) refers to the normalization applied after the residual connection. This attention mechanism implicitly integrates intra-view and cross-view interactions within the feature space, promoting complementary fusion and effective integration of multi-omics data. As a result, it improves the overall feature representation capability and enhances the model’s ability to capture complex multi-omics dependencies for survival prediction. A detailed analysis of the attention mechanism used in our model is provided in Section 12, available as supplementary data at *Bioinformatics* online.

### 2.5 Cancer survival prediction module

The core objective of survival prediction is to estimate the risk probability of an event occurring before a given point in time (Rai *et al.* 2021). To achieve this, the Cox proportional hazards model is widely used in survival analysis and effectively estimates the risk function of individuals at various time points. In this paper, we use the final fused feature $\bar{H}$ as the input to the Cox model and optimize the Cox loss function using the negative log partial likelihood function. Specifically, the Cox loss function can be expressed as:


(13) $$L_{\mathrm{cox}}=-\sum_{i=1}^{N}c_{i}[\beta^{T}{\bar{H}}_{i}-\log(\sum_{j\epsilon O(t_{i})}\;\text{exp}(\beta^{T}{\bar{H}}_{j}))]$$

where $c_{i}\in \{0,1\}$ represents the censoring status, $O(t_{i})$ is the set of individuals who were still in the risk concentration before time $t_{i}$.

In summary, the total loss function of model is given in Equation (14). We also outline the optimization process of SLCGF in Algorithm 1, available as supplementary data at *Bioinformatics* online


(14) $$L=L_{\mathrm{intra}}+L_{\mathrm{inter}}+L_{\mathrm{cox}}$$

### 2.6 Evaluation metrics

In this study, we use the concordance index (*C*-index) and the area under the curve (AUC) as evaluation metrics. The *C*-index and AUC are both widely used and crucial metrics for evaluating model performance in survival analysis (Wang *et al.* 2024). During the evaluation, any sample pair in which the earlier event is censored, or both samples are censored, will be excluded from the computation (Wen and Li 2023). The implementation details of the *C*-index and AUC are provided in Section 3, available as supplementary data at *Bioinformatics* online.

We employ five-fold cross-validation to comprehensively evaluate prediction performance. In each fold, models are trained on 80% of the data and evaluated on the remaining 20% held-out test set. The *C*-index and AUC are calculated on the test sets, and their mean and standard deviation are reported as the final results. For the independent KIRP test set, the trained models are directly applied, and the mean and standard deviation of the *C*-index and AUC are reported across five runs. All specific implementation details and parameter settings are provided in Section 4, available as supplementary data at *Bioinformatics* online, and the parameter sensitivity analysis can be found in Section 13, available as supplementary data at *Bioinformatics* online.

## 3 Results

### 3.1 Comparison results with baseline models

To conduct a more extensive comparative analysis, we compare our model with other advanced single-omics and multi-omics survival prediction methods, with single-omics methods including RSF (Ishwaran *et al.* 2008), En-cox (Yang and Zou, 2013), Deephit (Lee *et al.* 2018), and DeepSurv (Katzman *et al.* 2018), and multi-omics methods including HFBSurv (Li *et al.* 2022), CAMR (Wu *et al.* 2023), CustOmics (Benkirane *et al.* 2023), FGCNSurv (Wen and Li, 2023), and PCLSurv (Li *et al.* 2025). We report the average and standard deviation of the *C*-index and AUC obtained by each method in Table 2 and Table 3, available as supplementary data at *Bioinformatics* online, respectively. For single-omics methods, we provide experimental results using mRNA expression and microRNA data as input separately. Overall, SLCGF achieves the highest *C*-index on nine of 11 cancer datasets and the highest AUC on seven of 11 datasets, while ranking second on the remaining datasets under both evaluation metrics. For instance, on the Breast dataset, our method achieves *C*-index and AUC values of 0.838 and 0.863, respectively, achieving improvements of 6.1% and 4.8% over the second-best method PCLSurv. Unlike PCLSurv, which relies on *k*-means clustering to obtain prototypes, SLCGF adopts a similarity-based soft-label contrastive learning strategy that provides smoother and more fine-grained supervision for each sample pair. This design alleviates the limitations associated with low-quality prototypes and the absence of joint optimization, while more effectively capturing complex relationships in multi-omics data, leading to improved survival prediction performance. Furthermore, deep learning methods demonstrate superior performance in survival prediction tasks compared to traditional non-deep learning approaches. For example, DeepSurv consistently outperforms the traditional survival analysis method En-cox across multiple datasets, further confirming that deep learning frameworks effectively capture complex patterns and nonlinear relationships in data. From the perspective of multi-omics versus single-omics, we find that some multi-omics methods outperform single-omics methods in terms of performance. This suggests that complementary information from different omics layers provides more comprehensive feature representation, thus improving model performance. For example, our method, along with other multi-omics approaches such as PCLSurv and FGCNSurv, surpasses single-omics methods in terms of C-index and AUC values across most datasets. Notably, in some cases, single-omics methods perform better than multi-omics methods, which may be due to ineffective noise suppression in multi-omics data or the failure of omics data fusion to achieve the expected effect. To evaluate the generalization capability of all models, we apply each trained model directly to the independent KIRP dataset. The results indicate that SLCGF still outperforms the other baselines. Moreover, SLCGF also exhibits the highest performance on the LUAD dataset with four omics types. Experimental details are provided in Sections 5 and 6, available as supplementary data at *Bioinformatics* online, respectively. Together, these results confirm its superiority and reliability in cancer survival prediction tasks.

**Table 2.** Performance comparison of all methods in terms of *C*-index values.

Dateset	RSF	En-cox	Deephit	DeepSurv	HFBSurv	CAMR	CustOmics	FGCNSurv	PCLSurv	SLCGF
AML	0.635/0.663	0.653/0.560	0.613/0.580	0.638/0.593	0.650 ± 0.07	0.654 ± 0.05	0.609 ± 0.09	0.681 ± 0.04	**0.751** ± **0.04**	0.735 ± 0.02
Breast	0.712/0.561	0.745/0.670	0.590/0.569	0.640/0.497	0.677 ± 0.03	0.667 ± 0.10	0.685 ± 0.07	0.756 ± 0.06	0.777 ± 0.06	**0.838** ± **0.05**
Colon	0.517/0.516	0.433/0.548	0.552/0.528	0.504/0.504	0.773 ± 0.07	0.687 ± 0.17	0.597 ± 0.11	0.710 ± 0.10	0.780 ± 0.04	**0.821** ± **0.05**
GBM	0.520/0.534	0.537/0.534	0.533/0.536	0.553/0.562	0.563 ± 0.06	0.659 ± 0.06	0.569 ± 0.05	0.610 ± 0.03	0.654 ± 0.01	**0.660** ± **0.03**
Kidney	0.726/0.700	0.709/0.631	0.615/0.560	0.687/0.630	0.574 ± 0.03	0.702 ± 0.10	0.658 ± 0.15	0.770 ± 0.06	0.800 ± 0.08	**0.826** ± **0.04**
Liver	0.626/0.625	0.570/0.558	0.561/0.482	0.597/0.545	0.524 ± 0.06	0.625 ± 0.09	0.647 ± 0.06	0.652 ± 0.06	0.670 ± 0.04	**0.728** ± **0.03**
Lung	0.607/0.575	0.512/0.575	0.597/0.546	0.555/0.531	0.584 ± 0.08	0.651 ± 0.08	0.605 ± 0.09	0.605 ± 0.06	0.683 ± 0.04	**0.705** ± **0.03**
Melanoma	0.591/0.552	0.590/0.567	0.548/0.528	0.602/0.568	0.622 ± 0.03	0.604 ± 0.03	0.517 ± 0.04	0.640 ± 0.05	0.651 ± 0.03	**0.702** ± **0.03**
Ovarian	0.564/0.556	0.536/0.454	0.570/0.596	0.617/0.497	0.594 ± 0.02	0.645 ± 0.02	0.576 ± 0.05	0.609 ± 0.05	0.680 ± 0.03	**0.690** ± **0.02**
Sarcoma	0.689/0.670	0.596/0.606	0.610/0.506	0.647/0.667	0.626 ± 0.05	0.657 ± 0.03	0.550 ± 0.09	0.703 ± 0.05	**0.755** ± **0.04**	0.754 ± 0.02
Integrated	0.713/0.701	0.600/0.561	0.503/0.551	0.711/0.688	0.663 ± 0.01	0.686 ± 0.05	0.575 ± 0.06	0.715 ± 0.01	0.715 ± 0.01	**0.716** ± **0.01**

The best results are indicated in bold.

**Figure 2.**
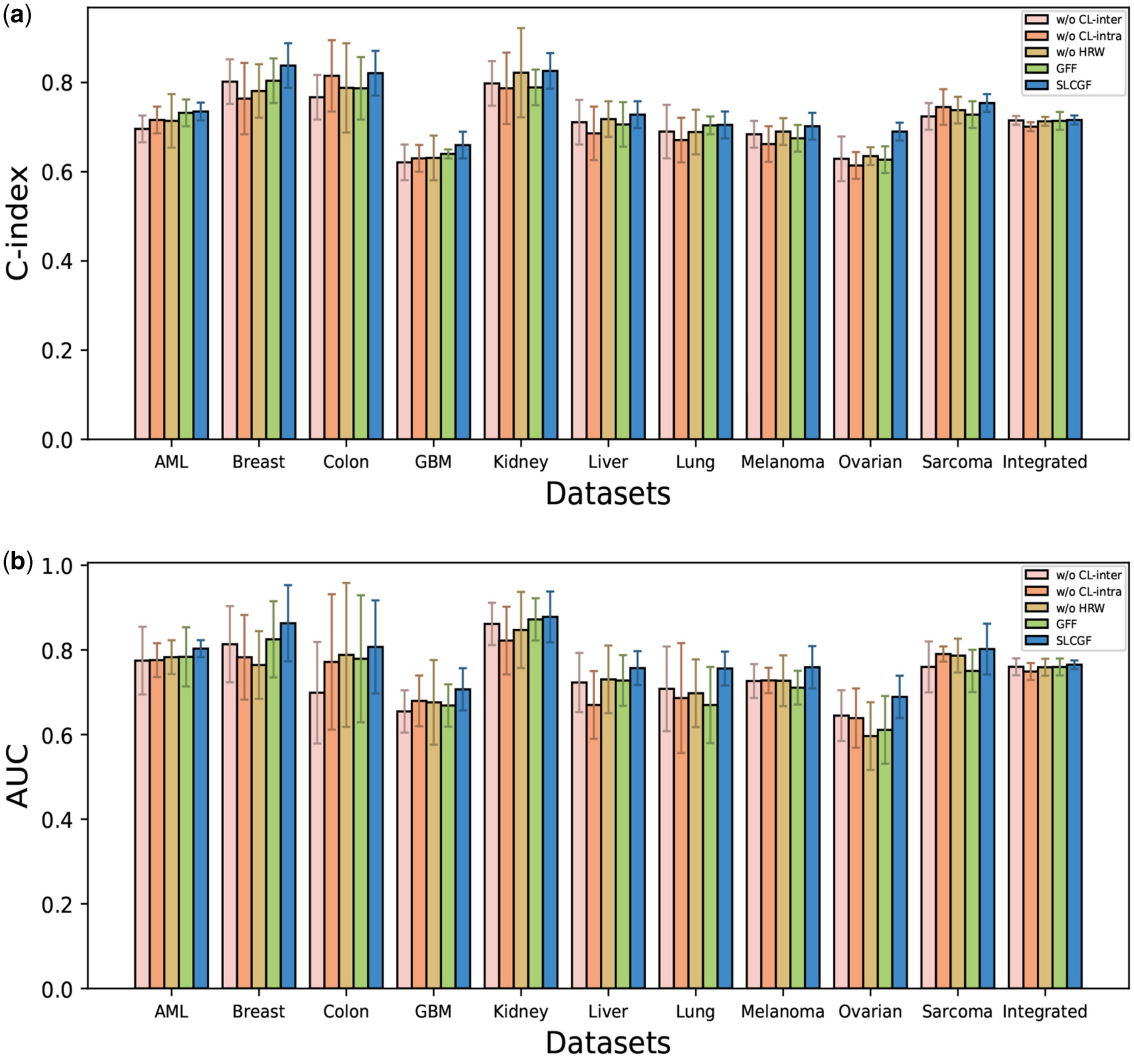
Ablation experiment results. (a) *C*-index values for each dataset obtained by different variant models. (b) AUC values for each dataset obtained by different variant models.

**Figure 3.**
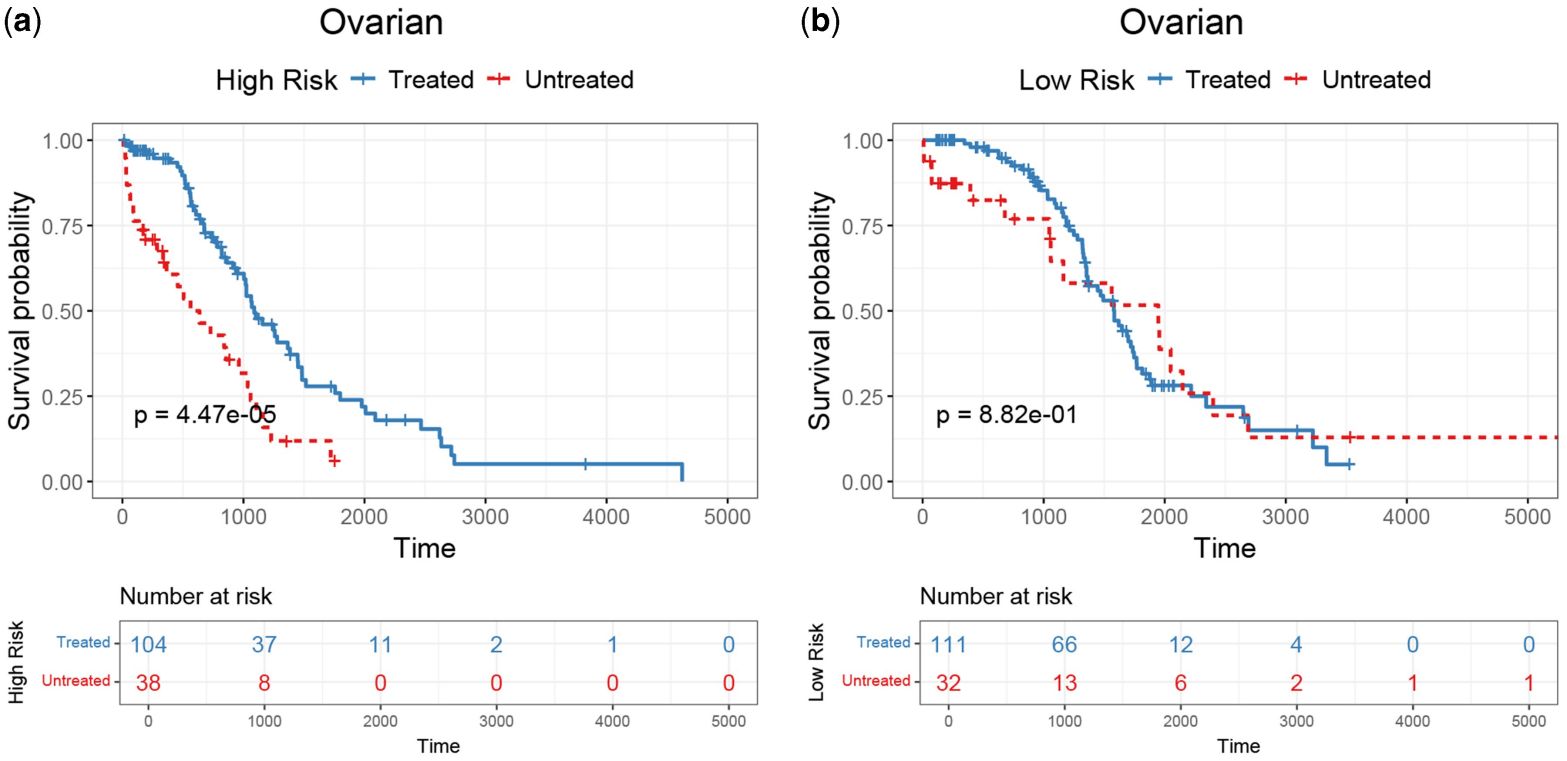
The impact of drug therapy on stratified patients. (a) The Kaplan–Meier curve of the impact of drug therapy on the survival of high-risk patients. (b) The Kaplan–Meier curve of the impact of drug therapy on the survival of low-risk patients.

To evaluate the survival differences between patients in different risk levels, we use the log-rank test method. Specifically, based on the predicted risk ratio from the model, we divide the patients into the high-risk and low-risk groups using the median risk ratio as the threshold. Then, we systematically compare the actual survival times of the two groups using the log-rank test. The lower the *P*-value, the better the model’s performance in risk stratification and prognostic prediction. As shown in Fig. 2, available as supplementary data at *Bioinformatics* online, we present the Kaplan–Meier curves of SLCGF and other comparison methods on the ovarian dataset. The results indicate that SLCGF achieves a significant *P*-value (*P*-value = 9.26e−06), effectively distinguishing between the high-risk and low-risk groups. In contrast, some methods, such as En-cox and DeepHit exhibit significant overlap in their predicted curves, failing to adequately differentiate between the high-risk and low-risk groups. This indicates that the risk scores predicted by our method have strong biological significance, providing a more accurate reflection of the patients’ prognostic risk and further validating the efficacy of our model. In addition, to comprehensively demonstrate the superiority of SLCGF across different datasets, we present its Kaplan–Meier curves across all datasets in Fig. 3, available as supplementary data at *Bioinformatics* online.

### 3.2 Univariate and multivariate cox proportional hazards analysis

To evaluate whether the predicted risk of SLCGF is significantly associated with survival, we perform univariate and multivariate Cox proportional hazards analyses, comparing the predicted risk of SLCGF with five clinical and pathological factors. These factors include age, clinical stage, neoplasm histologic grade, and tumor residual disease (Guo *et al.* 2015). The relevant experimental results are shown in Table 3. In the univariate Cox proportional hazards analysis, the predicted risk of SLCGF is significantly associated with survival (*P*-value <.005), with a hazard ratio (HR) of 1.99, indicating that the mortality risk for the high-risk group is significantly higher than that of the low-risk group. In the multivariate analysis, the predicted risk of SLCGF remains significant (*P*-value <.005), with the HR of 1.95. This indicates that even after controlling for other clinical variables, the model’s impact on survival remains significant, demonstrating its strong independent predictive ability. Overall, our model shows significant predictive power for survival.

**Table 3.** Hazard ratios of univariate and multivariate Cox proportional hazards analysis on the ovarian dataset.

		Univariate			Multivariate		
Variable		Hazard ratio	95% CI	*P*-value	Hazard ratio	95% CI	*P*-value
Age	≤50/>50	1.30	0.88–1.94	.19	1.20	0.80–1.80	.38
Stage	≤II/>II	2.47	0.91–6.66	.08	2.08	0.73–5.93	.17
Grade	≤G2/>G2	1.60	0.99–2.59	.06	1.36	0.82–2.26	.24
Tumor residual disease	≤1 cm/>1 cm	0.94	0.69–1.30	.72	0.89	0.64–1.24	.50
SLCGF	Low risk/high risk	1.99	1.46–2.72	<.005	1.95	1.42–2.67	<.005

### 3.3 Ablation study

In this section, to validate the effectiveness of the proposed modules, we conduct a series of ablation experiments to observe and analyze the impact of each module on the model’s performance. In these experiments, all parameter settings in the network model, except for the removed module, are kept consistent with the original experiment to ensure the comparability and consistency of the results. We compare the proposed model with four variants for analysis: w/o CL-inter, which removes the cross-view consistency loss; w/o CL-intra, which removes the intra-consistency loss from the contrastive learning process; w/o HRW, which replaces the matrix constructed using high-order random walks with an identity matrix; and GFF, which replaces the feature fusion module with a simple feature concatenation method.

To comprehensively evaluate the effects of different variants, we conduct ablation experiments on 11 datasets, assessing the C-index and AUC values. The experimental results are shown in Fig. 2. Specifically, after removing the intra-consistency loss, the performance of all datasets declines, with the most significant change observed in the breast dataset, where the *C*-index decreases by 7.4%. This result underscores the critical role of the intra-consistency loss in enhancing model performance. Upon removing the cross-view consistency loss, the model’s robustness significantly decreases, as this loss contributes to improving the complementarity of cross-view information, thereby optimizing multi-view information fusion. For instance, in the Colon dataset, removing this module leads to a decrease in the *C*-index from 0.821 to 0.767, with a notable decline in AUC values as well. Additionally, when the random walk module is removed, the *C*-index and AUC values for all datasets show a decline, indicating that the soft labels generated by random walks help the model better capture the relationships between different samples, thereby improving prediction performance. Finally, the feature fusion module consistently outperforms the direct feature concatenation method, particularly in the Ovarian and Melanoma datasets, where the performance improvement is most significant. This further confirms the positive impact of feature fusion on survival prediction tasks. To further verify the stability of the ablation results, we conduct multiple trainings of the full model and its variants under 10 different random seeds and calculate the mean and standard deviation of the *C*-index and AUC. We also perform paired *t*-tests to evaluate the statistical significance of performance differences between the proposed model and each ablated variant. As shown in Tables 4 and 5, available as supplementary data at *Bioinformatics* online, most datasets exhibit significant performance drops (*P*-value <.05) when any module is removed, highlighting the contribution of each model component. We also notice that there are a few exceptions. For instance, in the Kidney and Integrated datasets, removing the intra-view contrastive learning module has only a minor impact on performance, possibly because the feature distributions within each modality are relatively consistent and thus limiting the module’s contribution to enhancing discriminative power. Similarly, in the Lung dataset, replacing the fusion module with a simple concatenation operation still yields competitive performance. We hypothesize that the limited performance drop in this case may be attributed to low inter-modality heterogeneity or weak structural dependencies in the data, which collectively reduce the benefit of global feature fusion.

### 3.4 Impact of chemotherapy on survival prediction

In this experiment, we investigate the impact of chemotherapy on cancer patient survival of different risk groups. To this end, we first obtain the Carboplatin treatment information for patients from the TCGA database. Then, we divide the patients into two groups according to the predicted risk scores and investigate the impact of Carboplatin treatments on patients’ survival within each risk group. As shown in Fig. 3, for high-risk patients, the Kaplan–Meier curve results show that the survival time of patients who receive the drug treatment is significantly longer than those who do not undergo chemotherapy, highlighting the potential benefits of the treatment in improving survival outcomes (*P*-value = 4.47e−05). In contrast, for low-risk patients, the survival outcomes between the drug therapy and non-drug therapy groups do not differ substantially (*P*-value = 8.82e−01). While these findings suggest the potential necessity of stratified application of drug therapy, our analysis is based on retrospective data without control for confounding factors such as treatment timing and dosage. Therefore, further validation through prospective studies or more rigorous statistical control is needed to establish a causal relationship between risk scores and treatment efficacy.

## 4 Conclusion

In this article, we propose SLCGF, an innovative cancer survival prediction framework that combines soft-label guided contrastive learning and global feature fusion. By utilizing soft labels generated through random walk, the soft-label guided contrastive learning optimizes both intra-view and inter-view contrastive processes, thereby more precisely extracting the unique and shared information within omics data. Meanwhile, the global feature fusion module effectively captures the global interaction relationships in multi-omics data, significantly enhancing the model’s performance in survival prediction. Extensive experimental results demonstrate that SLCGF outperforms existing learning methods in survival prediction tasks.

Although SLCGF has achieved optimal prediction results, there is still room for further improvement. For example, incorporating pathology image modalities will significantly enhance the model’s predictive capability. Introducing explainable learning methods will help reveal the specific contributions of different omics features to the prediction, thereby increasing the model’s transparency and reliability. Future research will focus on integrating pathology images and explainable learning to further improve prediction performance and enhance the model’s credibility in clinical applications.

## Supplementary Material

btaf552_Supplementary_Data

## Data Availability

The datasets are publicly available and can be obtained from: https://github.com/alcs417/CGGA/tree/main/cancer_datasets and https://figshare.com/articles/dataset/MLOmics_Cancer_Multi-Omics_Database_for_Machine_Learning/28729127.
