# Supplementary material for: Cancer survival prediction based on soft-label guided contrastive learning and global feature fusion
Source: Bioinformatics. 2025 Oct 1;41(10):btaf552. doi: 10.1093/bioinformatics/btaf552 (PMC12548053; doi:10.1093/bioinformatics/btaf552)
Supplement: btaf552_Supplementary_Data [file btaf552_supplementary_data.pdf]

Supplementary File

Contents

1 Pseudocode of SLCGF 1

2 Summary of Cancer Datasets 2

3 Evaluation Metrics 3

4 Implementation details 4

5 Evaluation on an External Independent Test Set 5

6 Prediction Performance on the LUAD Dataset with Four Omics Types 6

7 Performance Comparison of Methods Based on AUC Values . . 7

8 Kaplan-Meier Curves 8

9 Comparison of Ablation Results Under Multiple Random Seeds 11

10 Impacts of Similarity Metrics 12

11 Impacts of Soft Label Construction Methods 13

12 Impacts of Self-attention Mechanism 14

13 Sensitivity Analysis 15

14 Effect of Batch Size on Soft Label Structure 17

References 17

1 Pseudocode of SLCGF

Algorithm 1 Pseudocode of SLCGF

Input: multi-omics dataset $X = \{X^1, X^2, ..., X^V\}$, censoring status c, observed time t, epochs T, temperature τ , step s, kernel temperature σ, balance weight γ

```
Initialize model parameters
for epoch=1 to T do
    Obtain the latent feature representations z_i^v, \hat{z}_i^v of each omics data by Eq.(2) and (3)
    Obtain the cross-view embedded features R_i^{v→m} for each omics by Eq.(4)
    Define soft labels by using random walk by Eq.(6)
    Compute the intra-view contrastive loss L_{intra} by Eq.(7)
    Compute the inter-view contrastive loss L_{inter} by Eq.(9)
    Obtain the fused multi-omics features \bar{H} by Eq.(12)
    Compute the Cox model loss L_{cox} by Eq.(13)
    Update the model parameters according to Eq.(14)
end for
```

Output: Survival risk score e

2 Summary of Cancer Datasets

Table S1. Summary of cancer datasets used in our study.

Cancer type	No. of samples	No. of mRNAs	No. of miRNAs	No. of CNV	No. of Methy
AML	160	2000	558	-	-
Breast	619	2000	891	-	-
Colon	219	2000	613	-	-
GBM	273	2000	534	-	-
Kidney	182	2000	796	-	-
Liver	365	2000	852	-	-
Lung	329	2000	878	-	-
Melanoma	428	2000	901	-	-
Ovarian	285	2000	616	-	-
Sarcoma	255	2000	838	-	-
Integrated	2557	2000	643	-	-
KIRP	272	2000	489	-	-
LUAD	441	2000	427	2000	2000

3 Evaluation Metrics

The C-index is primarily used to assess the concordance probability between the predicted survival time and the actual observed survival time. The C-index is defined as [1]:

$$\text{C-index} = \mathbb{P}\left(\beta^T \bar{H}_i > \beta^T \bar{H}_j \mid t_j > t_i, c_i = 1\right) \tag{1}$$

On the other hand, the AUC metric is used to evaluate the model's ability to discriminate individual event risks by assessing its performance in ranking. Specifically, AUC quantifies prediction accuracy by checking whether the predicted risk ordering of assessable pairs aligns with the actual order of event occurrences. The AUC is defined as [2]:

$$\text{AUC} = \frac{1}{\tilde{N}} \sum_{\mathbb{T} \in \mathcal{Y}} \sum_{i:t_i < \mathbb{T}} \sum_{j:t_j > \mathbb{T}} \mathbb{I}(\beta^T \bar{H}_i > \beta^T \bar{H}_j) \tag{2}$$

where $\mathcal{Y}$ denotes the set of all observed event times in the dataset, $\tilde{N}$ represents the cumulative number of assessable sample pairs computed over the entire event time set, and $\mathbb{I}$ denotes the indicator function. Both the C-index and AUC range from 0 to 1, with higher values indicating better predictive performance.

4 Implementation details

During the training process, we use the Adam optimizer with a weight decay of 1e-4 to train the model for 300 epochs, with a batch size of 64 and an initial learning rate of 0.002. For the hyperparameters in the experiment, the temperature τ in contrastive learning is set to 0.5, the step size s is set to 4, the kernel temperature σ is set to 0.9, and the balance weight γ is set to 0.5. Our experiments are conducted in a Python 3.7.12 and PyTorch 1.12.1 environment, with training and evaluation performed on an NVIDIA GeForce RTX 2080 Ti GPU. To conduct a more comprehensive comparative analysis, we evaluate other advanced single-modal and multi-modal methods. All comparative methods are applied to the same dataset, and we follow the default parameter settings recommended by the authors of each method.

5 Evaluation on an External Independent Test Set

To evaluate the cross-dataset generalization capability of all models, we directly apply each model trained from the original Kidney dataset on the external independent KIRP dataset. We also record the mean and standard deviation of the C-index and AUC reported across five runs. As shown in Figure S1, SLCGF still achieves better performance over existing baselines, demonstrating its robust generalization ability and reliability on independent datasets.

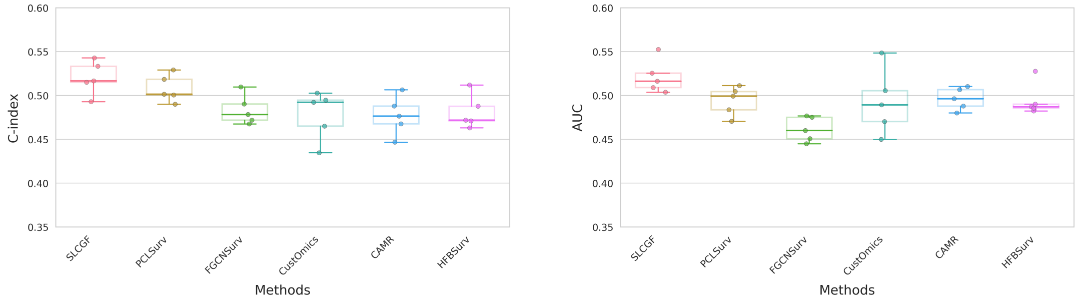

(a) **(b)**

Figure S1. Performance comparison of all models in terms of C-index and AUC on the external independent KIRP test set. Each method is performed five times to account for model randomness.

6 Prediction Performance on the LUAD Dataset with Four Omics Types

We conduct extended experiments on the LUAD dataset, which includes four omics types, to evaluate the applicability of SLCGF to general multi-omics datasets and to systematically compare it with other survival prediction methods. For methods that can only take a single modality as input, we apply them to each omics dataset separately and report their prediction results. All methods are evaluated using five-fold cross-validation and Table S2 demonstrates the mean and standard deviation of the C-index and AUC values. As expected, SLCGF outperforms the other baselines under both evaluation metrics, further validating its effectiveness in cancer survival prediction tasks by leveraging multi-omics datasets.

Table S2. Performance comparison of all methods in terms of C-index and AUC on the LUAD dataset with four omics data types.

Method	Modality	C-index	AUC
RSF	Gene Expression	0.545±0.05	0.551±0.06
	miRNA Expression	0.573±0.06	0.582±0.05
	CNV	0.521±0.05	0.515±0.08
	Methylation	0.484±0.03	0.461±0.06
En-cox	Gene Expression	0.575±0.05	0.586±0.06
	miRNA Expression	0.558±0.06	0.554±0.08
	CNV	0.493±0.06	0.487±0.07
	Methylation	0.529±0.06	0.535±0.06
DeepHit	Gene Expression	0.491±0.07	0.504±0.06
	miRNA Expression	0.518±0.06	0.531±0.09
	CNV	0.485±0.03	0.520±0.05
	Methylation	0.494±0.07	0.472±0.06
DeepSurv	Gene Expression	0.559±0.06	0.550±0.08
	miRNA Expression	0.493±0.06	0.489±0.06
	CNV	0.501±0.06	0.494±0.05
	Methylation	0.553±0.03	0.565±0.07
HFBSurv	All	0.651±0.06	0.669±0.04
CAMR	All	0.603±0.07	0.628±0.04
CustOmics	All	0.580±0.03	0.621±0.04
FGCNSurv	All	0.602±0.04	0.629±0.06
PCLSurv	All	0.624±0.06	0.640±0.04
Our Method	All	**0.657±0.03**	**0.689±0.03**

7 Performance Comparison of Methods Based on AUC Values

Table S3. Performance comparison of all methods in terms of AUC values.

Dateset	RSF	En-cox	Deephit	DeepSurv	HFBSurv	CAMR	CustOmics	FGCNSurv	PCLSurv	**SLCGF**
AML	0.640/0.684	0.662/0.616	0.638/0.626	0.678/0.619	0.656±0.03	0.659±0.04	0.611±0.14	0.738±0.08	**0.841±0.07**	0.803±0.02
Breast	0.627/0.562	0.685/0.639	0.562/0.569	0.663/0.536	0.690±0.09	0.717±0.02	0.776±0.09	0.754±0.11	0.815±0.05	**0.863±0.09**
Colon	0.447/0.463	0.631/0.689	0.572/0.558	0.546/0.598	0.743±0.06	0.696±0.25	0.667±0.12	0.712±0.13	0.753±0.14	**0.807±0.11**
GBM	0.443/0.506	0.582/0.608	0.590/0.533	0.550/0.569	0.592±0.02	0.650±0.06	0.605±0.07	0.665±0.05	**0.707±0.02**	**0.707±0.05**
Kidney	0.738/0.724	0.720/0.677	0.616/0.598	0.626/0.639	0.581±0.09	0.756±0.10	0.675±0.20	0.832±0.07	0.833±0.08	**0.878±0.06**
Liver	0.563/0.585	0.557/0.571	0.531/0.579	0.513/0.516	0.580±0.01	0.661±0.14	0.653±0.07	0.668±0.06	0.689±0.05	**0.757±0.04**
Lung	0.563/0.581	0.538/0.553	0.525/0.596	0.547/0.532	0.702±0.08	0.676±0.13	0.660±0.12	0.630±0.09	0.726±0.06	**0.756±0.04**
Melanoma	0.465/0.519	0.538/0.540	0.508/0.572	0.643/0.620	0.660±0.02	0.612±0.03	0.585±0.04	0.683±0.06	0.697±0.04	**0.759±0.05**
Ovarian	0.548/0.557	0.597/0.604	0.523/0.566	0.607/0.531	0.598±0.04	0.671±0.06	0.637±0.05	0.597±0.07	**0.729±0.05**	0.689±0.05
Sarcoma	0.658/0.606	0.593/0.544	0.647/0.569	0.604/0.608	0.593±0.04	0.643±0.04	0.582±0.14	0.754±0.07	**0.813±0.01**	0.802±0.06
Integrated	0.656/0.665	0.585/0.568	0.542/0.528	0.637/0.683	0.698±0.02	0.680±0.10	0.592±0.09	0.760±0.02	0.761±0.01	**0.765±0.01**

8 Kaplan-Meier Curves

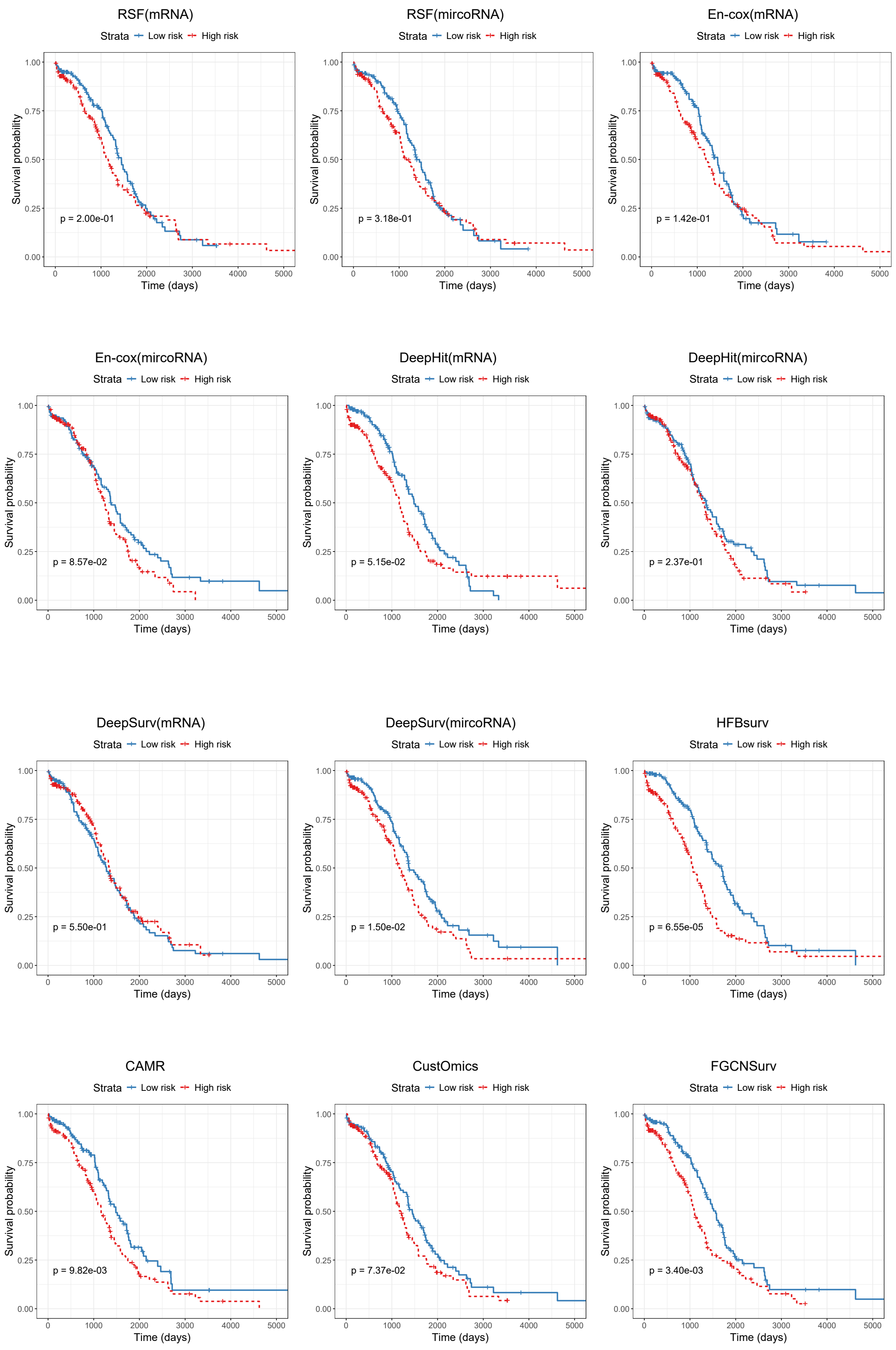

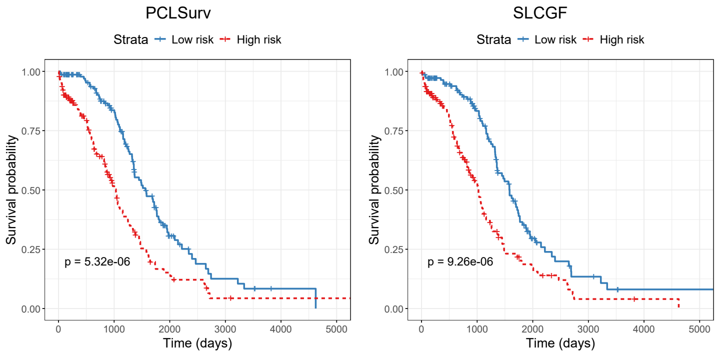

Figure S2. The Kaplan-Meier curves for high-risk and low-risk groups obtained from different survival prediction methods on the ovarian dataset.

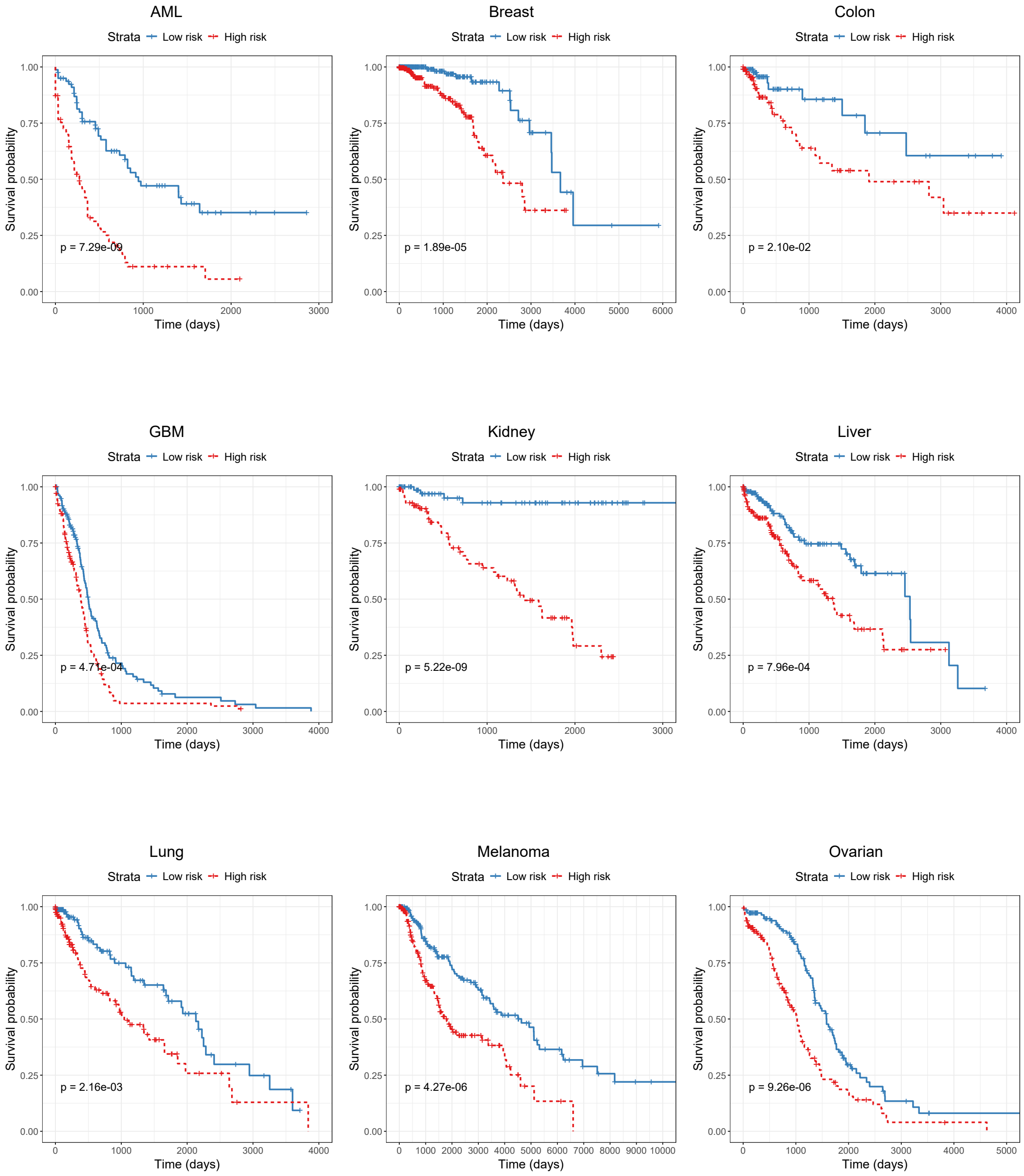

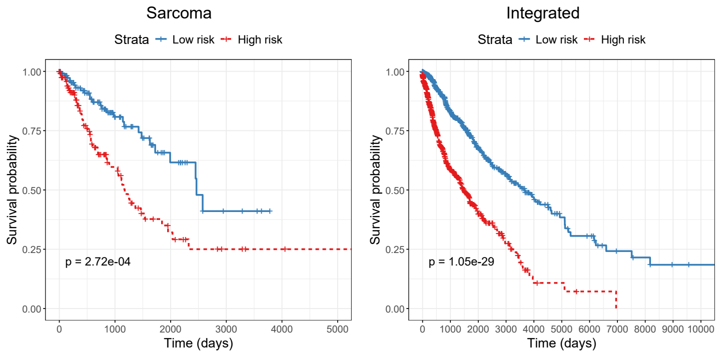

Figure S3. The Kaplan-Meier curves for high-risk and low-risk groups obtained by the SLCGF method on different datasets.

9 Comparison of Ablation Results Under Multiple Random Seeds

Table S4. Mean ± standard deviation of the C-index and paired t-test P-values for different model variants across all datasets averaged over multiple random seeds.

Dataset	w/o CL-inter		w/o CL-intra		w/o HRW		GFF		**SLCGF**
	Mean±Std	p-value	Mean±Std	p-value	Mean±Std	p-value	Mean±Std	p-value	Mean±Std
AML	0.700±0.02	0.002	0.698±0.01	0.005	0.702±0.02	0.007	0.709±0.01	3e-4	**0.740±0.01**
Breast	0.801±0.01	0.005	0.797±0.01	0.021	0.802±0.02	0.020	0.809±0.01	0.012	**0.824±0.02**
Colon	0.788±0.03	0.013	0.806±0.01	0.020	0.805±0.01	0.001	0.798±0.01	0.001	**0.829±0.01**
GBM	0.621±0.01	0.003	0.622±0.01	1e-5	0.613±0.02	0.002	0.619±0.01	3e-4	**0.647±0.01**
Kidney	0.804±0.02	**0.179**	0.795±0.01	0.026	0.794±0.02	0.045	0.797±0.01	0.043	**0.816±0.02**
Liver	0.705±0.01	4e-4	0.691±0.03	0.013	0.711±0.01	0.015	0.707±0.01	0.016	**0.731±0.01**
Lung	0.689±0.01	0.016	0.695±0.01	0.046	0.700±0.02	0.008	0.700±0.01	**0.144**	**0.712±0.01**
Melanoma	0.681±0.01	0.016	0.674±0.01	3e-4	0.683±0.01	0.008	0.679±0.01	8e-4	**0.706±0.01**
Ovarian	0.648±0.01	0.001	0.638±0.01	0.001	0.645±0.01	0.003	0.635±0.01	3e-4	**0.681±0.01**
Sarcoma	0.731±0.02	0.003	0.742±0.01	0.011	0.731±0.01	0.001	0.741±0.02	0.014	**0.756±0.01**
Integrated	0.707±0.01	**0.099**	0.697±0.01	0.001	0.700±0.01	0.027	0.706±0.01	0.037	**0.716±0.01**

Table S5. Mean ± standard deviation of the AUC and paired t-test P-values for different model variants across all datasets averaged over multiple random seeds.

Dataset	w/o CL-inter		w/o CL-intra		w/o HRW		GFF		**SLCGF**
	Mean±Std	p-value	Mean±Std	p-value	Mean±Std	p-value	Mean±Std	p-value	Mean±Std
AML	0.763±0.02	0.004	0.759±0.02	0.003	0.764±0.03	0.008	0.772±0.01	0.010	**0.811±0.02**
Breast	0.804±0.01	5e-4	0.810±0.02	0.001	0.790±0.02	2e-4	0.807±0.01	0.001	**0.850±0.02**
Colon	0.760±0.05	0.029	0.798±0.01	0.019	0.802±0.01	0.036	0.803±0.03	0.029	**0.827±0.03**
GBM	0.647±0.02	2e-5	0.659±0.02	0.001	0.670±0.02	0.002	0.668±0.02	0.019	**0.703±0.01**
Kidney	0.836±0.02	0.007	0.832±0.01	0.003	0.829±0.02	0.014	0.852±0.02	**0.061**	**0.863±0.02**
Liver	0.731±0.02	0.008	0.717±0.03	0.031	0.727±0.02	0.002	0.736±0.02	0.013	**0.761±0.01**
Lung	0.704±0.02	0.002	0.710±0.02	0.034	0.728±0.02	0.004	0.705±0.03	0.010	**0.757±0.02**
Melanoma	0.724±0.01	0.005	0.718±0.01	0.004	0.715±0.02	0.002	0.718±0.01	0.020	**0.739±0.01**
Ovarian	0.657±0.02	0.008	0.641±0.03	0.015	0.653±0.03	0.03	0.639±0.04	0.023	**0.696±0.02**
Sarcoma	0.768±0.03	0.009	0.783±0.01	0.007	0.790±0.02	0.024	0.787±0.02	0.023	**0.808±0.02**
Integrated	0.753±0.01	**0.066**	0.740±0.01	0.004	0.741±0.01	0.007	0.755±0.01	0.022	**0.763±0.01**

10 Impacts of Similarity Metrics

To validate the effectiveness of the selected heat kernel similarity, we further conduct ablation experiments by replacing it with Euclidean distance and cosine similarity. As shown in Table S6, the heat kernel similarity outperforms the other two similarity measures on most datasets, demonstrating its contribution in supporting robust graph learning for improved survival prediction.

Table S6. Model performance with different similarity measures in terms of AUC and C-index values across datasets.

Cancer Type	Metric	Heat Kernel Similarity	Cosine Similarity	Euclidean Distance
AML	AUC	**0.803±0.02**	0.770±0.02	0.769±0.07
	C-index	**0.735±0.02**	0.709±0.04	0.727±0.04
Breast	AUC	**0.863±0.09**	0.843±0.06	0.815±0.05
	C-index	**0.838±0.05**	0.824±0.04	0.817±0.04
Colon	AUC	0.807±0.11	**0.823±0.10**	0.801±0.10
	C-index	**0.821±0.05**	0.813±0.10	0.807±0.06
GBM	AUC	**0.707±0.05**	0.664±0.06	0.687±0.07
	C-index	**0.660±0.03**	0.632±0.03	0.650±0.04
Kidney	AUC	**0.878±0.06**	0.858±0.06	0.869±0.08
	C-index	**0.826±0.04**	0.811±0.06	0.815±0.06
Liver	AUC	**0.757±0.04**	0.719±0.10	0.735±0.05
	C-index	**0.728±0.03**	0.697±0.06	0.725±0.03
Lung	AUC	**0.756±0.04**	0.709±0.05	0.754±0.08
	C-index	**0.705±0.03**	0.696±0.03	0.703±0.04
Melanoma	AUC	**0.759±0.05**	0.747±0.03	0.746±0.02
	C-index	**0.702±0.03**	0.681±0.02	0.697±0.03
Ovarian	AUC	**0.689±0.05**	0.632±0.10	0.655±0.10
	C-index	**0.690±0.02**	0.653±0.05	0.659±0.03
Sarcoma	AUC	0.802±0.06	**0.819±0.02**	0.785±0.08
	C-index	**0.754±0.02**	0.751±0.03	0.753±0.03
Integrated	AUC	**0.765±0.01**	0.751±0.02	0.759±0.01
	C-index	**0.716±0.01**	0.701±0.01	0.706±0.01

11 Impacts of Soft Label Construction Methods

To validate the effectiveness of using the same soft labels in both intra-view and inter-view contrastive learning, we reconstruct the soft labels required for inter-view contrastive loss using the cross-view representation $R^{v \to m}$. As shown in Table S7, the unified soft label approach obtains better results across multiple datasets, ensuring consistency of supervisory signals and promoting stable optimization and feature alignment.

Table S7. Comparison of C-index and AUC values across datasets using different soft label construction methods.

Cancer Type	Metric	Our	$R^{v \to m}$
AML	AUC	**0.803±0.02**	0.758±0.09
	C-index	**0.735±0.02**	0.689±0.04
Breast	AUC	**0.863±0.09**	0.841±0.05
	C-index	**0.838±0.05**	0.823±0.06
Colon	AUC	**0.807±0.11**	0.693±0.18
	C-index	**0.821±0.05**	0.751±0.09
GBM	AUC	**0.707±0.05**	0.666 ±0.07
	C-index	**0.660±0.03**	0.639±0.04
Kidney	AUC	**0.878±0.06**	0.834±0.05
	C-index	**0.826±0.04**	0.799±0.04
Liver	AUC	0.757±0.04	**0.772±0.04**
	C-index	**0.728±0.03**	0.726±0.04
Lung	AUC	**0.756±0.04**	0.722±0.06
	C-index	0.705±0.03	**0.717±0.04**
Melanoma	AUC	**0.759±0.05**	0.739±0.05
	C-index	**0.702±0.03**	0.699±0.01
Ovarian	AUC	**0.689±0.05**	0.651±0.08
	C-index	**0.690±0.02**	0.660±0.02
Sarcoma	AUC	**0.802±0.06**	0.795±0.04
	C-index	**0.754±0.02**	0.739±0.04
Integrated	AUC	**0.765±0.01**	0.757±0.02
	C-index	**0.716±0.01**	0.712±0.02

12 Impacts of Self-attention Mechanism

We conduct a comparative analysis between the attention mechanism used in SLCGF and two other attention mechanisms, i.e. intra-view attention and cross-view attention. Specifically, for intra-view attention, we apply self-attention separately to each omics without considering cross-view interactions. For cross-view attention, we employ bidirectional modeling by alternately using each modality as the query and treating the others as key/value. We then replace our self-attention mechanism with each of the two attention strategies and conduct experiments on all datasets. As shown in Table S8, our approach consistently achieves superior performance except only one dataset, demonstrating its effectiveness in multi-modal fusion tasks.

Table S8. Performance comparison of different attention mechanisms in terms of AUC and C-index values.

Cancer Type	Metric	Our	Intra-view attention	Cross-view attention
AML	AUC	**0.803±0.02**	0.618±0.12	0.789±0.03
	C-index	**0.735±0.02**	0.565±0.06	0.726±0.03
Breast	AUC	**0.863±0.09**	0.653±0.16	0.828 ±0.07
	C-index	**0.838±0.05**	0.667±0.14	0.799±0.04
Colon	AUC	**0.807±0.11**	0.798±0.07	0.785±0.10
	C-index	**0.821±0.05**	0.769±0.05	0.815±0.07
GBM	AUC	**0.707±0.05**	0.653±0.09	0.699 ±0.06
	C-index	**0.660±0.03**	0.609±0.06	0.645±0.02
Kidney	AUC	**0.878±0.06**	0.699±0.09	0.841±0.06
	C-index	**0.826±0.04**	0.713±0.07	0.803±0.05
Liver	AUC	**0.757±0.04**	0.711±0.03	0.750±0.01
	C-index	**0.728±0.03**	0.679±0.05	0.719±0.02
Lung	AUC	**0.756±0.04**	0.626±0.07	0.722±0.10
	C-index	**0.705±0.03**	0.638±0.08	0.692±0.04
Melanoma	AUC	**0.759±0.05**	0.627±0.08	0.750±0.03
	C-index	**0.702±0.03**	0.595±0.04	0.692±0.02
Ovarian	AUC	**0.689±0.05**	0.635±0.05	0.651±0.05
	C-index	**0.690±0.02**	0.588±0.03	0.642±0.02
Sarcoma	AUC	0.802±0.06	0.597±0.18	**0.809±0.03**
	C-index	0.754±0.02	0.599±0.08	**0.758±0.03**
Integrated	AUC	**0.765±0.01**	0.570±0.09	0.759±0.01
	C-index	**0.716±0.01**	0.562±0.07	0.714±0.01

13 Sensitivity Analysis

We investigate the impact of the random walk step size s and the hyperparameter σ on the prediction results. Specifically, we analyze the variations in C-index and AUC values with step sizes ranging from 2 to 8 and σ values ranging from 0 to 1, as shown in Figure S4 and Figure S5. We can see that both the C-index and AUC values remain stable within a narrow range across different settings of s and σ. Taken together, these results confirm that our model is generally robust to varying hyperparameter settings and can be easily tuned to achieve competitive performance.

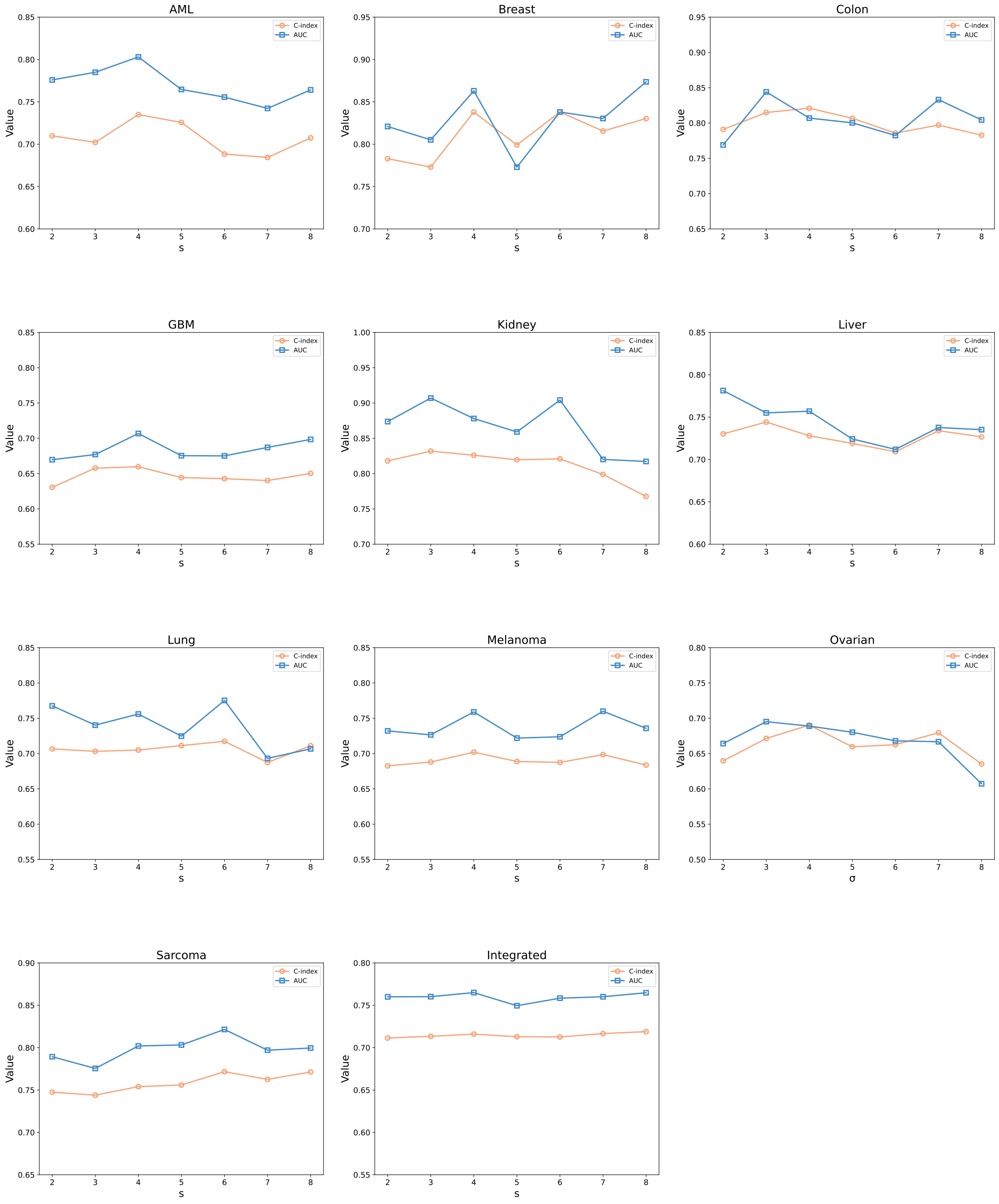

Figure S4. The C-index and AUC values for survival prediction with step sizes ranging from 2 to 8 across all datasets.

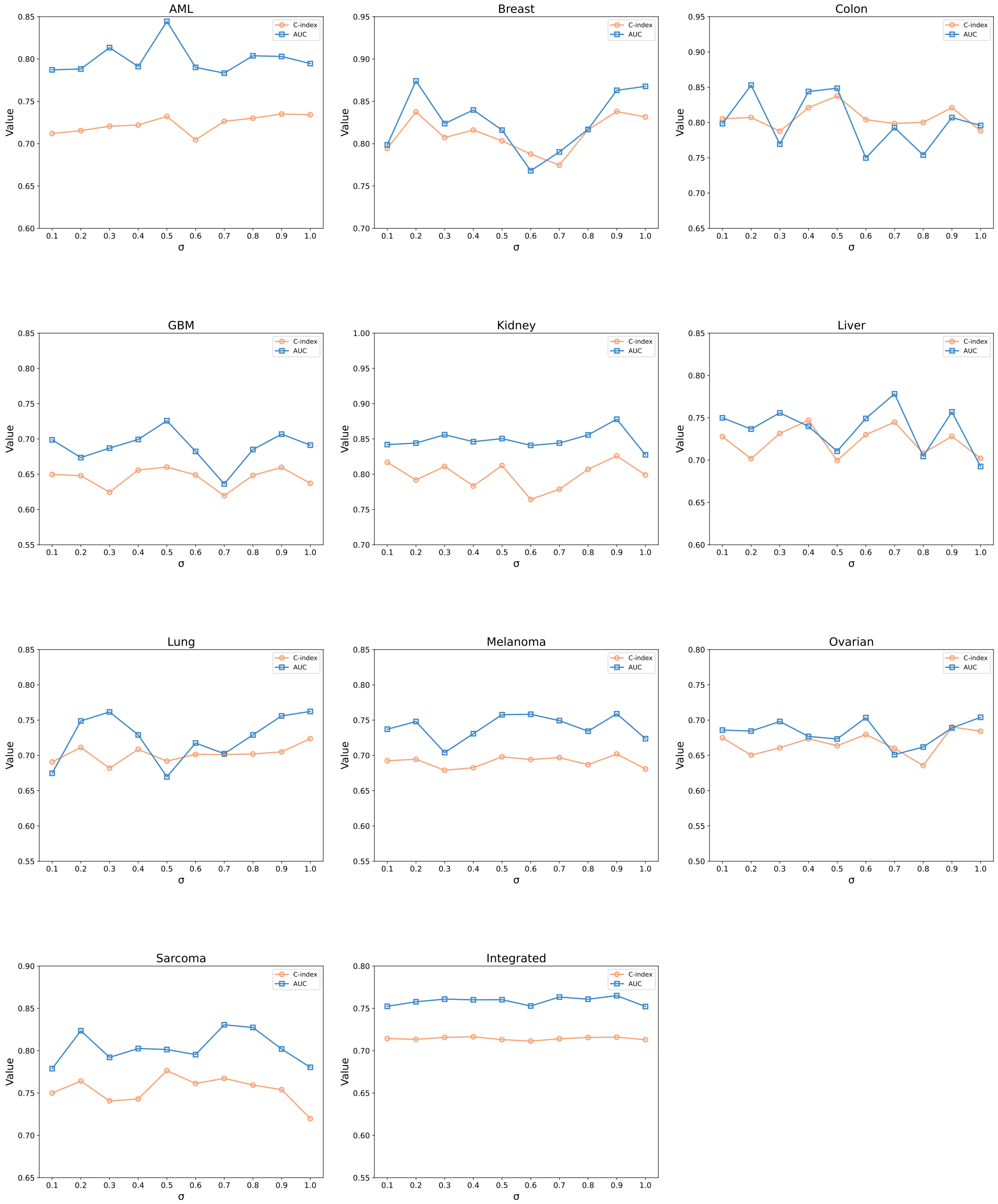

Figure S5. The C-index and AUC values for survival prediction with σ ranging from 0.1 to 1 across all datasets.

14 Effect of Batch Size on Soft Label Structure

To investigate whether different batch sizes affect the generated soft labels, we conduct a visualization analysis on the integrated dataset that includes eight distinct cancer types. Specifically, we first generate soft-label matrices with batch sizes of 64 and 128, respectively, and then apply spectral clustering on each matrix, followed by t-SNE visualization in two-dimensional space. As shown in Figure S6, when the batch size is set to 64, the cluster structure is more compact, and the boundaries between clusters are clearly distinguishable. In contrast, with a batch size of 128, we observe noticeable color mixing within certain clusters. These results suggest that larger batch sizes may weaken the model's ability to capture fine-grained differences between samples, leading to less discriminative soft labels, whereas smaller batch sizes facilitate learning clearer similarity structures and producing more separable soft labels.

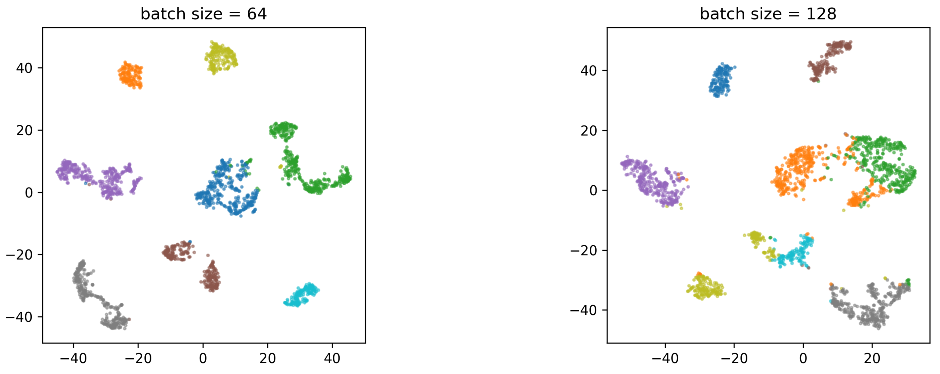

(a) (b)

Figure S6. Visualization of soft label structures generated with batch sizes of (a) 64 and (b) 128 on the integrated dataset.

References

[1] Mayr A and Schmid M. Boosting the concordance index for survival data–a unified framework to derive and evaluate biomarker combinations. *PloS one*, 9(1):e84,483, 2014.

[2] Wen G and Li L. FGCNSurv: dually fused graph convolutional network for multi-omics survival prediction. *Bioinformatics*, 39(8):btad472, 2023.
